# Supplementation of the freezing extender with glutathione peroxidase improves the post-thaw quality of ram semen by enhancing antioxidant capacity, regulating metabolic enzyme activity, and modulating proteome expression

**DOI:** 10.5713/ab.250985

**Published:** 2026-05-07

**Authors:** Kexin Wang, Qian Zhou, Yuping Wang, Zhixing Shen, Jun Li, Xue Li, Shengrong Shi, Yining Yan, Chunxiang Zhang, Shi Lei, Youshe Ren

**Affiliations:** 1 College of Animal Science, Shanxi Agricultural University, Taigu, China; 2 Shanxi Key Laboratory of Animal Genetics Resource Utilization and Breeding, Jinzhong, China

**Keywords:** Antioxidant, Glutathione Peroxidase, Proteomics, Ram, Semen Cryopreservation

## Abstract

**Objective:**

The present study was conducted to determine effects of different concentrations of glutathione peroxidase (GSH-Px) on sperm motility and viability, structural integrity, antioxidant capacity, and metabolic enzyme activity after freezing semen of sheep and to evaluate the expression of differential proteins.

**Methods:**

Semen samples were collected from six Dorper rams, pooled, and subsequently divided into four groups. GSH-Px was added to the cryoprotectant solution at concentrations of 0, 0.8, 1.6, and 3.2 units per liter, respectively. Following thawing, sperm motility, viability, and kinematic parameters were assessed, along with acrosomal and plasma membrane integrity, DNA integrity, mitochondrial activity, antioxidant status, metabolic enzyme activity, and proteomic profiles.

**Results:**

Adding 1.6 U/L GSH-Px to the freezing extender significantly enhanced the motility and viability of sperm after freeze-thaw, as well as the integrity of the acrosome, plasma membrane and DNA. The activities of total antioxidant capacity (T-AOC), superoxide dismutase and catalase were significantly increased. The oxidative stress marker malondialdehyde was significantly reduced, and the activities of metabolic enzymes including lactate dehydrogenase, aspartate aminotransferase, and alanine aminotransferase were also decreased. Proteomic analysis identified 150 differentially expressed proteins. Western blotting demonstrated the increase G1 to S Phase Transition 1, ribosomal protein L11, and downregulated annexin A2 expression.

**Conclusion:**

Supplementing the cryopreservation extender with 1.6 U/L GSH-Px significantly improves post-thaw ovine sperm quality, as evidenced by enhanced structural integrity, elevated T-AOC, and attenuated oxidative stress-induced damage.

## INTRODUCTION

Semen preservation is a critical component of artificial insemination (AI) technology, serving as a fundamental basis for the efficient utilization of superior genetic resources [1]. Semen cryopreservation technology, as a primary method of *in vitro* preservation, offers distinct advantages over room temperature and low-temperature preservation techniques. This approach enables AI to transcend temporal and spatial limitations, enhances the utilization efficiency of superior breeding males, and promotes the accelerated genetic improvement of high-quality animal genetic resources [2]. The ongoing advancement of semen cryopreservation techniques has been instrumental in facilitating the development and application of AI.

However, during semen cryopreservation, the rapid temperature decline can impair the antioxidant enzyme system in seminal plasma, which plays a crucial role in maintaining reactive oxygen species (ROS) homeostasis. Under ultra-low temperature conditions (−196°C), antioxidant enzyme activity is markedly suppressed, resulting in the accumulation of excessive ROS generated by sperm metabolism and the disruption of redox balance. This oxidative stress may compromise sperm motility and fertilization potential [3]. Such damage compromises both membrane integrity and functional competence, ultimately resulting in reduced semen quality [4,5]. Therefore, the selection of optimal antioxidants and cryoprotectants is essential for mitigating oxidative damage during semen cryopreservation, preserving sperm motility, and maintaining overall semen quality by preventing sperm injury caused by oxidative stress.

The glutathione peroxidase (GSH-Px) family, recognized as a critical antioxidant enzyme system, is widely distributed in eukaryotic cells. It functions as a key enzyme in the degradation of hydrogen peroxide (H_2_O_2_) and represents one of the primary defense mechanisms against peroxide-induced toxicity [6,7]. GSH-Px employs selenocysteine (Sec) as a catalytic residue, enabling the rapid catalysis of hydrogen peroxide reduction and the efficient utilization of glutathione (GSH) as a reducing substrate [8]. The enzyme also facilitates the reduction of hydrogen peroxide or organic peroxides to water or their respective alcohols, thereby contributing to significant antioxidant activity [9,10]. These research findings indicate that GSH-Px is crucial to the antioxidant defense system and plays a significant role in mitigating membrane damage caused by ROS [11]. Previous studies have demonstrated that eight glutathione peroxidase isozymes (GPx1 to GPx8) have been identified in mammals, each exhibiting distinct physiological functions [12,13]. Specifically, glutathione peroxidase 1 (GSH-Px1) modulates the insulin signaling pathway; glutathione peroxidase 3 (GPX3) is most widely distributed and has been detected in the proximal convoluted tubules of the kidney, plasma, body fluids, and even in the male reproductive tract and sperm; glutathione peroxidase 4 (GPX4) is a crucial component of the mitochondrial sheath in mammalian spermatozoa [14]; glutathione peroxidase 5 (GPX5), an epididymis-specific GSH-Px, plays a significant role in maintaining redox homeostasis by scavenging ROS within the epididymal lumen [15]. Collectively, GSH-Px-mediated antioxidant defense is essential for sperm functional integrity and the maintenance of a reduced microenvironment conducive to sperm maturation. Previous studies have demonstrated that the addition of GSH-Px to the freezing extender significantly improves outcomes in boar and canine semen cryopreservation [16,17].

To elucidate the protective mechanism of GSH-Px supplementation in frozen-thawed ram sperm, this study assessed sperm quality, antioxidant parameters, and seminal plasma metabolic enzyme activities, combined with proteomic validation, to provide a theoretical foundation for its application in semen preservation.

## MATERIALS AND METHODS

### Animals management and semen collection

This experiment was conducted at the Sheep Farm Experimental Station of Shanxi Agricultural University, spanning the summer to autumn season from May 20 to July 20 (latitude 37.42°N, longitude 112.58°E). Six healthy 3-year-old Dorper rams were selected for the study. During the semen collection period, the rams were housed individually and provided with an additional egg and one carrot as part of their daily feed ration. In addition, each ram was allowed at least thirty min of free activity daily (established feeding guidelines of the Beijing Agricultural Data Information Center). Semen was collected twice weekly using the artificial vagina method, with a minimum interval of two days between consecutive collections, and the total number of collections did not exceed 40. Freshly collected semen samples were evaluated immediately for quality. Only samples meeting the following criteria were included in the study: semen volume >0.5 mL, sperm concentration ≥2×10^9^ sperm/mL, and total sperm motility exceeding 80%.

### Semen diluent and cryopreservation procedure

Each mixed semen sample was divided into four equal aliquots and extended with cryopreservation diluents containing 0, 0.8, 1.6, and 3.2 U/L of GSH-Px. The experiment consisted of four groups: one control group supplemented with GSH-Px-free cryoprotectant, and three treatment groups supplemented with 0.8, 1.6, and 3.2 U/L of GSH-Px (EC No. 1.11.1.9; MCE). The freezing extender was composed of Tris (2.7 g), citric acid (1.375 g), fructose (0.96 g), trehalose (1.6 g), streptomycin and penicillin solution (100 IU), 25% (v/v) egg yolk, and 3% (v/v) glycerol. The samples were cooled in a refrigerator at 4°C. After reaching 4°C, the semen was transferred into 0.25 mL straws, sealed with polyvinyl alcohol powder, and placed horizontally in a freezer container positioned 5 cm above the liquid nitrogen surface for 8 min. Finally, the straws were rapidly immersed in liquid nitrogen for long-term storage. All frozen samples were stored in liquid nitrogen for at least one month before quality assessment and experimental analysis. After thawing, six frozen straws (n = 6) were randomly selected from each treatment group to evaluate sperm motility, viability, and kinematic parameters. Unless otherwise stated, all reagents were obtained from Solarbio.

### Analysis of post-thaw sperm kinematics using a computer-assisted sperm analysis system

After thawing, the semen was mixed with a preheated base diluent. A 10 μL aliquot of the semen sample was transferred onto a pre-warmed slide maintained at 37°C and covered with a coverslip. Sperm motility and viability were assessed using a computer-assisted sperm analysis (CASA) system (SpermClass Analyser; Microptic SL), with measurements including curvilinear velocity (VCL, μm/s), straight-line velocity (VSL, μm/s), and average path velocity (VAP, μm/s). For each sample, at least five random fields of view were selected, and the experiment was repeated three times to calculate the mean of the replicate measurements.

### Sperm membrane functionality, DNA integrity, mitochondrial activity and acrosome integrity

#### Sperm plasma membrane integrity

The hypoosmotic swelling test (HOST) was conducted to assess the functional integrity of the sperm plasma membrane [18]. Following thorough mixing of the thawed semen, 20 μL was transferred into a centrifuge tube, to which 200 μL of hypoosmotic solution was gradually added. The mixture was incubated in a water bath at 37°C for 30 min. Thereafter, 10 μL of the suspension was placed onto a glass slide, allowed to air dry, and examined under a light microscope at 400× magnification. Sperm exhibiting curved tails were considered to have intact plasma membranes, whereas those in a naturally stretched configuration were indicative of compromised membrane integrity. The percentage of sperm with intact plasma membranes was calculated accordingly.

#### Assessment of sperm DNA integrity

The integrity of sperm DNA was evaluated using acridine orange/ethidium bromide (AO/EB) dual-fluorescence staining [19]. In brief, 65 μL of semen was combined with 30 μL of basic diluent, and 3 μL of AO followed by 4 μL of EB were added sequentially under gentle vortexing. The mixture was incubated in the dark at room temperature for 15 min, after which 8 μL of Hancock’s solution was introduced and thoroughly homogenized. An 8 μL aliquot was transferred onto a glass slide, gently covered with a coverslip, and examined under a fluorescence microscope at 400× magnification. For each sample, at least 400 spermatozoa were randomly selected and evaluated; spermatozoa exhibiting green fluorescence were classified as having intact DNA, whereas those showing red or yellow fluorescence were considered indicative of DNA damage or abnormalities.

#### Evaluation of sperm mitochondrial activity

The JC-1/PI double fluorescence staining method was used. Briefly, 30 μL of sample was mixed with 25 μL of basic diluent, and immediately supplemented with 2 μL of JC-1 (Catalog no. HY-15534; MCE) dye and 8 μL of PI (Catalog no. HY-D0815; MCE). The mixture was vortexed and incubated at 37°C in the dark for 30 min. Then, 8 μL of Hancock’s solution was added, and subsequently, 8 μL of the resulting mixture was placed on a glass slide for fluorescence microscopy at 400× magnification. At least 400 sperm per sample were analyzed. Sperm with a green head and orange tail show high mitochondrial activity. A dim or non-fluorescent tail indicates low mitochondrial activity.

#### Assessment of sperm acrosome integrity rate

Acrosome integrity was evaluated using chlortetracycline (CTC) fluorescence staining. A 20 μL aliquot of semen was combined with 20 μL of CTC staining solution, vortexed to ensure homogeneity, and incubated in the dark at room temperature for 3 min. Subsequently, 8 μL of 12.5% glutaraldehyde solution was added as a fixative, mixed thoroughly, and 4 μL of the resulting suspension was transferred onto a glass slide for examination under a 400× fluorescence microscope. A minimum of 400 spermatozoa were assessed across five standardized microscopic fields (four corners and central region). Spermatozoa with intact acrosomes exhibited bright, uniform yellowish-green fluorescence distributed throughout the entire head, whereas those with compromised acrosomal integrity displayed partial or complete absence of fluorescence.

### Determination of antioxidant capacity and metabolic enzyme activity of the semen of sheep after freeze-thaw

Frozen straws were retrieved from the liquid nitrogen tank and thawed in a 37°C water bath. Following thawing, samples were labeled according to their concentration groups and centrifuged at 4°C and 3,000×g for 15 min. Collect the supernatant for further analysis. The antioxidant capacity and metabolic enzyme activities were determined using commercial test kits (Nanjing Jiancheng Institute of Bioengineering). The tests were conducted in accordance with the instructions for superoxide dismutase (SOD) (A001-3-2), catalase (CAT) (A007-1-1), malondialdehyde (MDA) (A003-1) and total antioxidant capacity (T-AOC) (A015-2-1). The determination of metabolic enzyme activity was carried out in accordance with the instructions of lactate dehydrogenase (LDH) (A020-2-2), alanine aminotransferase (ALT) (C009-2-1), and aspartate aminotransferase (AST) (C010-2-1). MDA levels were measured using the thiobarbituric acid (TBA) method. Briefly, MDA reacts with TBA to form a red chromogenic product, which was generated by incubating the sample at 95°C for 40 min. Absorbance was measured at 532 nm, and results were expressed as nmol/mL. T-AOC was assessed using the ABTS method: samples were mixed with ABTS^+^ substrate and allowed to react at room temperature for 6 min. Absorbance was read at 425 nm, and activity was reported in units per liter (U/L). SOD activity was determined based on its ability to inhibit the reduction of WST-1 by superoxide anions. The reaction was carried out at 37°C for 20 min, and absorbance was measured at 450 nm. LDH activity was assayed according to the Reitman-Frankel principle: LDH catalyzes the conversion of lactate to pyruvate, which subsequently reacts with 2,4dinitrophenylhydrazine (DNPH) to form a red-brown hydrazone compound. Absorbance was measured at 440 nm, and enzyme activity was expressed in U/L. ALT and AST activities were also determined using the Reitman-Frankel colorimetric method. In both cases, pyruvate generated during the enzymatic reaction forms a red-brown phenylhydrazone with DNPH under alkaline conditions. Absorbance was measured at 505 nm for ALT and at 510 nm for AST. To ensure accuracy and reliability, strict quality control procedures were implemented. All samples were analyzed in triplicate for both technical and biological replicates. Each microplate included standard curve wells, blank control wells, and quality control sample wells as specified by the kit protocol. Quantitative calculations were based on standard curves generated using the kit-provided standards. Sample absorbance values were converted into concentration or activity units using these standard curves. Only data from batches where the linear regression coefficient (R^2^) of the standard curve exceeded 0.99 and the quality control sample measurements fell within the predefined acceptable range were included in the final analysis.

### Proteomic analysis of frozen-thawed sperm

#### Extraction of sperm proteins

Samples were weighed and subsequently transferred into 2 mL centrifuge tubes. Steel balls, lysis buffer (comprising 8 M urea and 50 mM Tris-HCl), along with Roche protease inhibitor cocktail (1×) were then added. Tubes were incubated on ice for 5 min, then homogenized at 60 Hz for 2 min using a tissue lyser. After centrifugation at 20,000×g for 15 min at 4°C, supernatants were collected. Dithiothreitol (DTT) was added to a final concentration of 10 mM, followed by incubation at 37°C for 1 h. Iodoacetamide (IAA) was then added to 20 mM and the samples were incubated in the dark for 30 min. The protein concentrations were measured using a Bio-Rad DC Protein Assay kit (Catalog no. P1200; Solarbio) following the manufacturer’s instructions [20].

#### Protein enzymatic hydrolysis and tandem mass tag labeling technology

A 150 μg aliquot of protein solution was mixed with trypsin at a 50:1 mass ratio and incubated at 37°C for 14–16 h to facilitate enzymatic digestion. The resulting peptides were desalted using a waters solid-phase extraction column and subsequently dried by vacuum centrifugation. For tandem mass tag (TMT) labeling, an aliquot of peptides was resuspended in 100 mM triethylammonium bicarbonate buffer (TEAB) buffer to a final volume of 30 μL. The TMT reagent was dissolved in 100% acetonitrile and added to the peptide solution at a 5:1 reagent-to-peptide mass ratio. The mixture was allowed to react at room temperature for 1–2 h. The reaction was terminated by adding 5% hydroxylamine to a final concentration of 0.4%. All labeled samples were pooled, homogenously mixed, vacuum-dried, and stored under –80°C for subsequent analysis.

#### Protein identification and LC-MS/MS analysis

Equal amounts of peptides from all samples were pooled, diluted with mobile phase A (5% acetonitrile, pH 9.8), and subjected to high-pH reversed-phase liquid chromatography using a 4.6×150 mm Agilent ZORBAX 300 Extend-C18 column (3.5 μm). Elution was performed at a flow rate of 0.3 mL/min with a gradient increasing from 5% to 90% mobile phase B (97% acetonitrile, pH 9.8) over 70 min. Elution peaks were monitored at 214 nm, and fractions were collected at 1-min intervals. Based on the chromatographic profile, 10 distinct fractions were consolidated, then lyophilized. The dried peptides were resuspended in 0.1% formic acid, centrifuged at 20,000×g for 10 min, and the supernatant was analyzed by nano-electrospray ionization (nanoESI) coupled with an Orbitrap Exploris 480 mass spectrometer (Thermo Fisher Scientific). Data-dependent acquisition (DDA) was performed for MS/MS analysis.

### Western blot analysis

Equal amounts of protein (60 μg) in each sample were separated and transferred onto nitrocellulose (NC) membranes by 10% or 12% sodium dodecyl sulfate-polyacrylamide gel electrophoresis (SDS-PAGE). Block the membrane with rapid protein blocking solution at room temperature for 15 min to reduce non-specific binding, then incubate overnight at 4°C and gently shake with the stock solution antibodies against G1 to S Phase Transition 1 (GSPT1) (1:500; Wuhan Aibotaike Company), Annexin A2 (ANXA2) (1:1,000; Wuhan Aibotaike Company), and Ribosomal Protein L11 (RPL11) (1:500; Wuhan Aibotaike Company). After three washes with Tris-Buffered Saline with Tween-20 (TBST) buffer (8 min per wash), the membranes were incubated with the secondary antibody IRDye 800 CW goat anti-rabbit immunoglobulin G (IgG) (1:20,000, D80426-08; LI-COR Bioscience) at room temperature for 90 min. Subsequently, the membranes were washed three times with TBST buffer (8 min per wash), and protein bands were visualized using an Odyssey infrared imaging system (LI-COR Biosciences). Gray-scale analysis was done using ImageJ.

### Statistical analysis

One-way analysis of variance (ANOVA) was used to compare differences among groups. When a significant overall effect was detected (p<0.05), post hoc multiple comparisons were conducted using Tukey’s honestly significant difference (HSD) test. Data were presented as mean±standard error of the mean (SEM). A p value less than 0.05 was considered statistically significant. For proteomics data, protein identification and quantification were performed based on TMT labeling and mass spectrometry data using MaxQuant software (ver. 2.1.4.0; https://www.maxquant.org/), with searches conducted against the UniProt database for the relevant species. Differentially expressed proteins (DEPs) were identified based on the following criteria: p<0.05 and a fold change greater than 1.2. To explore potential protein-protein interaction (PPI) networks, interactions were retrieved from the STRING database (https://string-db.org/, ver. 11.5) and analyzed using Cytoscape software, with the network visualized accordingly. Subsequent bioinformatics analyses were carried out in the R programming environment. Subcellular localization of DEPs was predicted using the WoLF PSORT tool (https://wolfpsort.hgc.jp/). Additionally, heatmap generation were performed using the OmicStudio online platform (https://www.omicstudio.cn/tool).

## RESULTS

### Analysis of sperm motility patterns by computer-assisted sperm analysis system

As shown in Table 1, adding different concentrations of GSH-Px to the cryoprotectant has a significant impact on the quality parameters of sperm after thawing. In terms of sperm viability, the 1.6 U/L GSH-Px treatment group was significantly higher than the control group and other treatment groups (p<0.05), but there was no significant difference between it and the 0.8 U/L group. In terms of sperm motility, the 1.6 U/L treatment group was significantly higher than the other groups (p<0.05). The analysis of movement parameters showed that the VAP and VSL of the 0.8 U/L and 1.6 U/L treatment groups were significantly higher than those of the other groups (p<0.05); among them, the VCL of the 1.6 U/L group was significantly better than the other groups (p<0.05).

### Analysis of sperm structural integrity system

The results in Table 2 show that adding GSH-Px to the cryopreservation dilution can improve the membrane integrity of sheep sperm after thawing. Compared with the control group, the 0.8 U/L and 1.6 U/L GSH-Px treatment groups could significantly improve the sperm’s plasma membrane integrity, DNA integrity, acrosome integrity and mitochondrial activity (p<0.05); among them, the 1.6 U/L treatment group performed the best in all indicators, significantly higher than other concentration groups (p<0.05). However, when the GSH-Px concentration increased to 3.2 U/L, its protective effect on the various structural integrity indicators significantly weakened, and there was no significant difference compared with the control group (p>0.05).

### Analysis of the antioxidant capacity and metabolic enzyme activity of sperm

**Figure 1:**
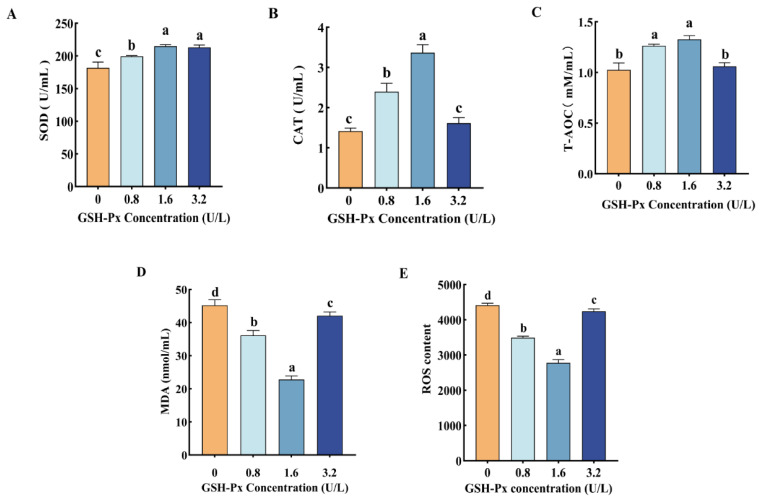
The effect of GSH-Px on the antioxidant status of thawed male sheep sperm. ^a–d^ Different superscript letters show statistically differences (p<0.05). SOD, superoxide dismutase; GSH-Px, glutathione peroxidase; CAT, catalase; T-AOC, total antioxidant capacity; MDA, malondialdehyde; ROS, reactive oxygen species.

As shown in Figure 1, compared with the control group, the addition of 0.8, 1.6 and 3.2 U/L GSH-Px significantly increased the activity of SOD, CAT and reduced the concentration of MDA and ROS (p<0.05). Compared with the control group, the SOD activity in the 1.6 and 3.2 U/L groups was significantly increased (p<0.05), but no significant difference was detected between these two experimental groups (p>0.05). Compared with the control group and the 3.2 U/L group, the T-AOC in the 0.8 U/L and 1.6 U/L groups was significantly increased (p<0.05).

**Figure 2:**
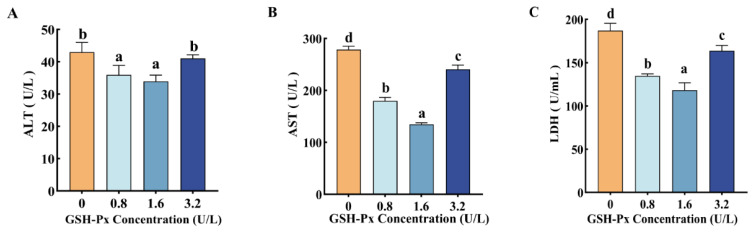
The influence of GSH-PX on the activities of enzymes related to energy metabolism in male semen and seminal plasma. ^a–d^ Different superscript letters show statistical differences (p<0.05). ALT, alanine aminotransferase; GSH-Px, glutathione peroxidase; AST, aspartate transaminase; LDH, lactate dehydrogenase.

Figure 2 shows the enzyme activities related to sperm metabolism after cryopreservation. Compared with the control group, adding 1.6 U/L GSH - Px to the cryopreservation diluent can significantly reduce the activities of ALT, LDH, and AST (p<0.05).

### Identification of differentially expressed proteins and research on their interaction networks

**Figure 3:**
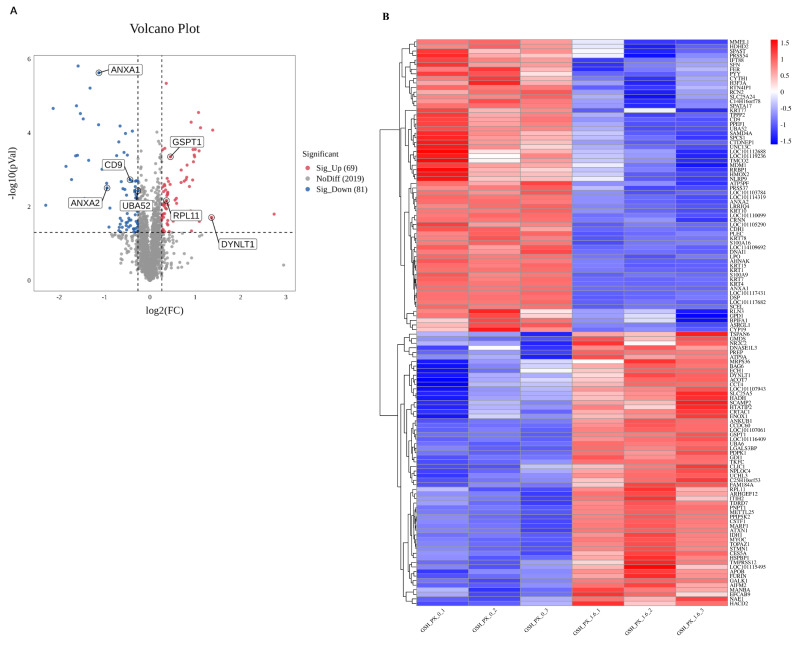
Differential expression and clustering analysis of proteins in ram sperm between control and GSH Px treated groups. (A) Volcano plot of differentially expressed proteins in ram sperm. (B) Heatmap of cluster analysis of differentially expressed proteins in ram sperm. Volcano plot (A): The x axis represents log_2_ (ratio) of the comparison group, which symmetrizes the ratio distribution. log_2_ (ratio)>0 indicates up regulated proteins, and log_2_ (ratio)<0 indicates down regulated proteins. Statistical significance was set at p<0.05. Color code red: significantly up regulated proteins (n = 69); blue: significantly down regulated proteins (n = 81); grey: non significant proteins (n = 2,019). Labeled proteins: ANXA1, CD9, UBA52, RPL11 and GSPT1 are listed as candidate key proteins (see text for details). Heatmap (B): Each row represents a protein, and each column represents an individual sperm sample. Sample groups (horizontal axis). GSH Px 0: control group; GSH Px 1.6: experimental group. The protein labeled in Figure 3A is a key candidate protein. ANXA1, annexin A1; CD9, cluster of differentiation 9; RPL11, protein L11; GSH-Px, glutathione peroxidase.

**Figure 4:**
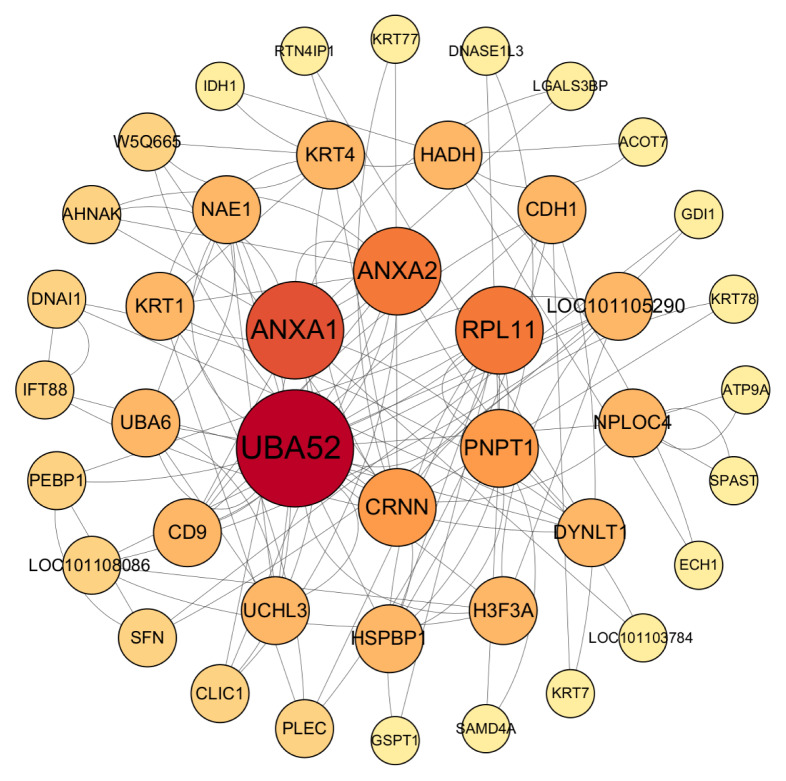
PPI network of DEPs in sheep sperm. Each circular node represents a DEPs. Node color intensity ranges from light yellow to dark red, reflecting the magnitude of differential expression, with darker shades indicating greater significance. PPI, protein-protein interaction; DEPs, differentially expressed proteins.

Proteomic analysis was performed on sperm samples cryopreserved from the experimental group, supplemented with 1.6 units/L of GSH-Px, and the control group, which lacked GSH-Px. As illustrated in Figures 3A, 3B, a total of 2,169 sperm proteins were identified, with 150 exhibiting differential expression. Among these DEPs, 81 were downregulated and 69 were upregulated. The PPI network presented in Figure 4 indicates that these DEPs do not function in isolation but instead form a tightly interconnected functional module centered on core proteins such as ubiquitin A-52 residue ribosomal protein fusion product 1(UBA52) and ANXA2.

#### Functional enrichment analysis of differentially expressed proteins

**Figure 5:**
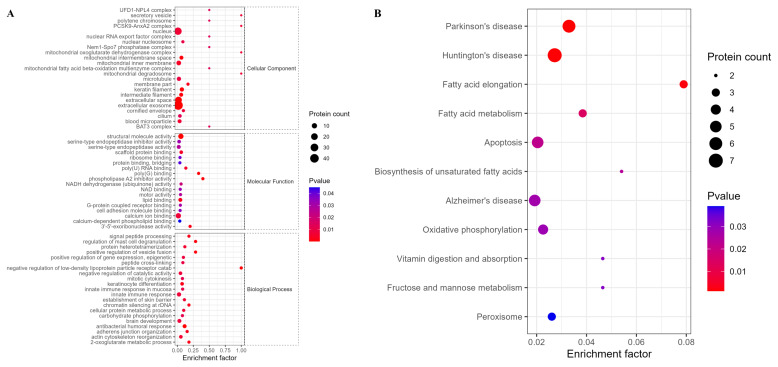
GO classification map of different proteins (A), scatter plot of the KEGG enrichment pathways (B). The biological processes and pathways in which the proteins of interest are involved are shown. GO, Gene Ontology; KEGG, Kyoto Encyclopedia of Genes and Genomes.

To achieve a more comprehensive understanding of the biological significance of DEPs, Gene Ontology (GO) and Kyoto Encyclopedia of Genes and Genomes (KEGG) pathway enrichment analyses were conducted. The GO analysis results reveal that DEPs are distributed across three primary functional domains: molecular function, cellular component, and biological process. Notably, the most significant enrichment is observed within the cellular component category (Figure 5A). The number of proteins associated with cellular components was higher than those involved in molecular functions and biological processes. Within the molecular function category, DEPs were enriched in such as structural constituent of sperm flagellum, calcium ion binding, DNA binding, and lipid binding. Additionally, KEGG pathway analysis identified 11 significantly enriched pathways (Figure 5B), including those associated with Parkinson’s disease, fatty acid elongation and metabolism, apoptosis, oxidative phosphorylation, vitamin digestion and absorption, fructose and mannose metabolism, and peroxisome function.

#### Validation of results via western blotting

**Figure 6:**
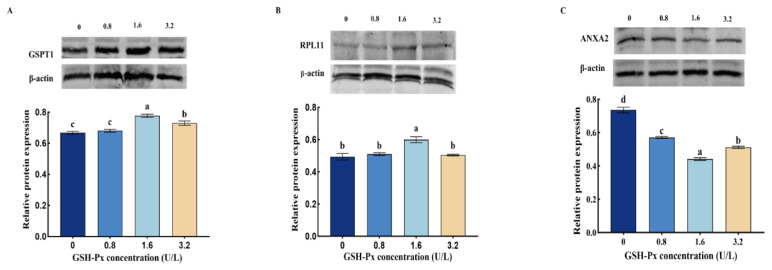
Western blot analysis for GSPT1 (A), RPL11 (B) and ANXA2 (C) of frozen-thawed ram spermatozoa in extenders supplemented with 0 and 1.6 U/L GSH-Px. ^a–d^ Different superscript letters show statistical differences (p<0.05). GSPT1, G1 to S phase transition 1; RPL11, ribosomal protein L11; ANXA2, annexin A2; GSH-Px, glutathione peroxidase.

The expression of GSPT1, RPL11 and ANXA2 in ram sperm was verified by Western blotting (Figure 6). As shown in Figures 6A, 6B, the expression levels of GSPT1 and RPL11 in the sperm of the 1.6 U/LGSH-Px experimental group were significantly higher than those in the control group (p<0.05). As shown in Figure 6C, the expression level of ANXA2 in sperm of the GSH-Px experimental groups were all significantly lower than that of the control group (p<0.05). It is notable that among these groups, the expression level of ANXA2 in the 1.6 U/L GSH-Px group was significantly lower than other groups (p<0.05).

## DISCUSSION

Enzymatic antioxidants, for example SOD and GSH-Px, play a critical role in maintaining redox homeostasis in sperm by neutralizing ROS and preventing oxidative damage [21]. Given the critical role of antioxidant enzymes in preserving semen quality, this study is the first to systematically investigate the effect of adding GSH-Px to the cryopreservation formula for ram semen. Compared with the control group, adding 1.6 U/L GSH-Px can significantly enhance sperm motility and sperm viability, improve sperm DNA integrity and plasma membrane integrity, and simultaneously enhance mitochondrial activity. However, no statistically significant difference was observed between the high-concentration group at 3.2 U/L and the control group. This outcome may be attributed to the accelerated consumption of GSH induced by high concentrations of GSH-Px. If GSH replenishment or its biosynthetic pathway cannot keep pace with demand, GSH levels may decline. Insufficient GSH can impair sperm energy metabolism and key enzyme activities, thereby negatively affecting sperm motility [22]. In the experimental group supplemented with 1.6 U/L GSH-Px, the activities of SOD and CAT were notably enhanced in comparison to those seen in the control group. Concurrently, levels of MDA in sperm from this group was significantly reduced. The observed decrease in MDA content may be attributed to the ability of GSH-Px to reduce and decompose lipid peroxides. By inhibiting lipid peroxidation reactions on the sperm membrane surface, GSH-Px effectively reduces MDA formation in seminal plasma [23]. Additionally, supplementation with GSH-Px led to a significant reduction in the activities of ALT, AST, and LDH. This observation aligns with previous studies on semen preservation, where decreased release of intracellular metabolic enzymes reflects improved integrity and stability of the sperm plasma membrane, underscoring the critical role of redox homeostasis in maintaining sperm structural integrity [20,24].

Based on the volcano plot of proteomics data, the analysis of PPI and a comprehensive analysis of other data, this study identified seven candidate proteins that may be involved in the regulation of the mechanism for cryopreservation of ram sperm: dynein light chain Tctex-type 1 (DYNLT1), cluster of differentiation 9 (CD9), GSPT1, RPL11, UBA52, annexin A1 (ANXA1) and ANXA2. These candidate proteins are involved in multiple key biological processes essential for spermatogenesis and sperm function, covering distinct aspects including sperm motility, oocyte recognition and binding, acrosome reaction, capacitation, and gene expression regulation. For instance, the DYNLT1 protein is localized in the sperm tail, where it functions as a component of cytoplasmic dynein motor protein complexes involved in intracellular motility [25]. CD9 is a critical molecule essential for sperm-oocyte binding and plays a pivotal role in regulating multiple biological processes underlying sperm-oocyte interactions. Moreover, CD9 participates in the regulation of sperm capacitation and the acrosome reaction, underscoring its multifunctional role in the fertilization process [26]. ANXA1 regulates the secretion of steroid hormones and is highly expressed in the testes and ovaries. Through experiments on male sperm, it has been found that ANXA1 may regulate the expression at the translational level during sperm production and improve the quality of sperm DNA [27,28]. UBA52 is a fusion protein consisting of an N-terminal ubiquitin moiety and a C-terminal ribosomal protein L40 [29], however, its specific role in sperm physiology remains to be elucidated.

To further clarify the freezing preservation effects of these candidate proteins after the addition of GSH-Px, we selected ANXA2 and RPL11, which are located at the center of the protein interaction network, and GSPT1, which is located at the outermost layer of the network, for experimental verification based on the topological structure of the PPI network and the differential expression ratios of the volcano plot. Western blot analysis revealed that, compared to the control group, the expression level of GSPT1 was significantly elevated in the GSH-Px experimental group, which is consistent with its established role as a critical regulator of cell survival and its involvement in fundamental cellular processes such as cell cycle regulation [30]. Furthermore, experimental with 1.6 U/L GSH-Px significantly upregulated the expression of ribosomal protein RPL11, which plays a pivotal role in ribosome biogenesis, mRNA processing, and DNA repair mechanisms [31,32]. In addition, the expression of ANXA2 was significantly downregulated, suggesting that the improvement in sperm plasma membrane integrity observed after freeze-thawing in the GSH-Px experimental group might be attributed to the maintenance of ANXA2 protein levels during cryopreservation.

The results demonstrated that the addition of GSH-Px to the freezing extender not only enhanced the antioxidant capacity of sperm and reduced oxidative stress to maintain their function, but also regulated the expression of key proteins related to cell survival and energy metabolism specifically, it upregulated GSPT1, RPL11, and downregulated ANXA2.

## CONCLUSION

This study demonstrates that the addition of 1.6 U/L GSH-Px to the cryoprotectant effectively preserves the quality of frozen-thawed ram sperm by enhancing antioxidant capacity, improving energy metabolism, and modulating the expression of key proteins.

## Figures and Tables

**Table 1:** The effect of supplementing varying concentrations of glutathione peroxidase (GSH-Px, U/L) on sperm parameters following freeze-thaw cycles

Items	Control	0.8	1.6	3.2	p-values
Sperm viability (%)	82.83±0.02^b^	87±0.01^ab^	90±0.01^a^	83.67±0.01^b^	0.001
Sperm motility (%)	43.63±0.55^c^	47.17±0.66^b^	59.64±0.68^a^	40.42±0.63^d^	<0.001
VAP (μm/s)	49.52±2.48^bc^	53.35±1.63^ab^	57.51±0.60^a^	45.78±0.90^c^	<0.001
VSL (μm/s)	28.33±0.89^b^	33.12±0.49^a^	37.83±0.21^a^	28.54±0.46^b^	<0.001
VCL (μm/s)	84.46±2.28^b^	87.86±0.79^b^	97.49±0.24^a^	77.53±0.51^c^	<0.001

Results are presented as mean±standard error.

a–d Different letters among the same group indicate significant differences between groups (p<0.05; Tukey HSD test).

The p-value indicates the significance level of the differences among the groups.

VAP, average path velocity; VSL, straight linearvelocity; VCL, curvilinear velocity; HSD, honestly significant difference.

**Table 2:** The effect of supplementing different concentrations of GSH-Px (U/L) on the structural integrity of sperm after freeze-thaw cycles

Items (%)	Control	0.8	1.6	3.2	p-values
HOST	47.60±0.45^c^	52.99±0.28^b^	56.21±0.46^a^	46.95±0.34^c^	<0.001
DNAI	45.78±0.47^d^	52.66±0.21^b^	57.57±0.47^a^	47.56±0.55^c^	<0.001
CTC	48.02±0.26^d^	54.87±0.35^b^	58.72±0.26^a^	50.94±0.39^c^	<0.001
JC	46.20±0.68^c^	53.05±0.57^b^	58.45±0.47^a^	48.37±0.95^c^	<0.001

Results are presented as mean±standard error.

a–d Different letters among the same group indicate significant differences between groups (p<0.05; Tukey HSD test).

The p-value indicates the significance level of the differences among the groups.

GSH-Px, glutathione peroxidase; HOST, plasma membrane integrity; DNAI, DNA integrity; CTC, acrosomal integrity; JC, mitochondrial activity; HSD, honestly significant difference.

## Data Availability

Upon reasonable request, the datasets of this study can be available from the corresponding author.
